# Sex Differences in the Epidemiology, Risk Factors, and Prognosis of Malignant Ventricular Arrhythmias in Sepsis Patients

**DOI:** 10.31083/j.rcm2504132

**Published:** 2024-04-03

**Authors:** Le Li, Xi Peng, Likun Zhou, Zhuxin Zhang, Yulong Xiong, Zhenhao Zhang, Zhao Hu, Yan Yao

**Affiliations:** ^1^Cardiac Arrhythmia Center, Chinese Academy of Medical Sciences, Peking Union Medical College, National Center for Cardiovascular Diseases, Fuwai Hospital, 100037 Beijing, China

**Keywords:** ventricular arrhythmias, epidemiology, risk factor, outcome, sex

## Abstract

**Background:**

Women are frequently underrepresented in clinical trials and databases focusing on ventricular arrhythmias (VAs). However, understanding sex-based differences in risk factors and the prognosis of VAs is essential for tailoring personalized prevention and treatment strategies. This study aimed to investigate sex differences in the epidemiology, risk factors, and prognosis of VAs in patients with sepsis.

**Methods:**

We conducted a comprehensive analysis of 27,139 sepsis patients (mean [SD] age, 66.6 [16.2] years; 15,626 [57.6%] male), among whom 1136 (4.2%) developed VAs during their hospitalization. We evaluated VAs incidence and potential risk elements in both male and female patients, along with in-hospital mortality.

**Results:**

Men had a significantly higher likelihood of developing VAs compared to women (odds ratio [OR]: 1.70, 95% confidence interval [CI]: 1.50–1.94, *p* < 0.001). In the case of non-ischemic cardiomyopathy (NICM), the association with VAs was stronger in men than in women (relative risk ratio [RRR] = 1.63, 95% CI: 1.10–2.40, interaction *p* = 0.014). Furthermore, we observed significant sex-specific interactions in the relationship between incident VAs, congestive heart failure (CHF) (RRR = 1.35, 95% CI: 1.03–1.76, interaction *p* = 0.031), and pneumonia (RRR = 1.33, 95% CI: 1.02–1.74, interaction *p* = 0.036) when considering the adjusted model. The presence of VAs was associated with a nearly twofold increase in the risk of in-hospital mortality, a result that was observed in both sexes.

**Conclusions:**

In sepsis patients, the emergence of VAs independently escalates the risk of in-hospital mortality, with a notable correlation between male sex and an increased VAs risk. The impacts of CHF, NICM and pneumonia on incident VAs were significantly influenced by sex.

## 1. Introduction

Infection induced-sepsis is prevalent in Intensive Care Units (ICU) and gives rise to numerous complications. Notably, it significantly impacts the heart. Sepsis-induced cardiac dysfunction extend beyond systolic and diastolic anomalies, encompassing cardiac rhythm disturbances [1]. While prior research has identified sepsis as a predisposing factor for arrhythmias, the emphasis has largely been on atrial arrhythmias [2]. Notably, malignant ventricular arrhythmias (VAs)—encompassing ventricular tachycardia (VT) and ventricular fibrillation (VF)—are of particular concern due to their potential to induce acute heart failure and result in sudden cardiac death (SCD) [3, 4]. Studies indicate that patients with sepsis are vulnerable to suffering from VAs [3]. These irregular heart rhythms can arise from factors such as electrolyte imbalances, reduced afterload, ventricular dysfunction, elevated catecholamines, and chronotropic dysregulation [5, 6].

While the relationship between sepsis and malignant VAs has been well investigated, the impact of sex differences is less explored. Previous studies have underscored sex-specific variations in the epidemiology, underlying mechanisms, clinical presentations, and outcomes of arrhythmias [7, 8, 9]. However, a significant portion of the evidence shaping clinical decisions in the field of cardiology is derived from studies with a conspicuous lack of female representation. Moreover, the sex-specific distribution of diseases, clinical risk elements, and outcomes of VAs in septic patients have been scarcely addressed. Gaining insights into how sex modulates the risk and prognosis of arrhythmias in these patients could lay the groundwork for personalized therapeutic approaches. With this in mind, we aimed to conduct a thorough assessment of sex disparities in the epidemiology, risk factors, and outcomes of VAs in septic patients.

## 2. Materials and Methods

### 2.1 Source of Data

We employed data from a substantial intensive care database, namely the Medical Information Mart for Intensive Care IV (MIMIC-IV, version 2.0). This repository includes data on more than 200,000 patients who were admitted to a variety of ICUs at Beth Israel Deaconess Medical Center (BIDMC) during the period from 2008 to 2019 [10]. It provides details on patient demographics, vital statistics, common health conditions, and lab results. The researcher (LL) has the necessary permissions to retrieve data from this database (record ID: 35965741). Given that our research involved a third-party, anonymized, and publicly accessible database with prior institutional review board (IRB) consent, there was no need for additional IRB approval from our side.

### 2.2 Study Population and Outcomes

This study encompassed individuals aged 18 and older who were primarily admitted to the hospital for sepsis. Those with missing or incomplete records were not included. For the purposes of our research, sepsis was characterized as organ dysfunction due to infection, defined by a rise in the Sequential Organ Failure Assessment (SOFA) score by at least 2 points [11]. The primary outcome was the incidence of VAs, encompassing both sustained and non-sustained VT, as well as VF, during the hospital stay. Guidelines provide specific details on the criteria for VT/VF [12].

### 2.3 Data Collection

For data retrieval, we utilized PostgreSQL tools (version 13.0, University of California, California, USA), employing unique patient identifiers, or Subject IDs, to accurately identify individual patients. Our focus was on collecting potential VT/VF risk factors for septic patients from their initial hospital visits. Conditions like congestive heart failure (CHF), atrial fibrillation (AF), non-ischemic cardiomyopathy (NICM), old myocardial infarction (OMI), acute myocardial infarction (AMI), and chronic kidney disease (CKD) have been linked with a heightened risk of VAs or SCD [13, 14]. Moreover, elements such as admission type, logistic organ dysfunction system (LODS) rating, length of stay in the ICU (LOS-ICU), occurrence of pneumonia, serum white blood cell (WBC) count, and the administration of specific medications (like macrolides, quinolones, vasoactive substances, or anti-arrhythmic drugs including amiodarone, propafenone, sotalol, and dronedarone)—vital for evaluating and managing sepsis—were factored into our analyses [6, 11]. For instances where multiple LODS score and WBC count records existed, we opted for the earliest entries. The specifics of these parameters are detailed in **Supplementary Table 1**. Information regarding in-hospital deaths, interpreted as mortality from any cause during hospital or ICU stay, was also sourced from the database.

### 2.4 Statistical Analysis

The Kolmogorov-Smirnov test was utilized to evaluate the normality of continuous variables. Continuous variables that adhered to a normal distribution were presented as mean ± standard deviation (SD), while those that did not follow a normal distribution were described using the median and interquartile range. Categorical information was depicted in terms of counts and proportions. Continuous variables were analyzed using the *t*-test or Welch’s *t*-test, while categorical variables were assessed with chi-squared analysis. Cumulative incidence graphs for VAs were generated, with death in the absence of VAs considered as a competing event. We used the odds ratio (OR) in conjunction with a 95% confidence interval (CI) to examine the associations between the variables under investigation and VAs, as well as the relationship between VAs and in-hospital mortality. In assessing potential risk determinants and VAs, a sex-based interaction was incorporated into the model, allowing the impact of the variable to differ by sex. All time-related models utilized age as the time metric [15].

To discern the relationships between potential risk determinants and VAs in both male and female sepsis patients, sex-segregated multivariable logistic analyses were conducted. Each risk determinant underwent a univariate logistic regression assessment. Furthermore, a comprehensive model encompassing admission type, LOS-ICU, LODS rating, CHF, AF, AMI, OMI, NICM, CKD, pneumonia, vasoactive drugs, antibiotics, and WBC was developed. Sex interactions were incorporated for all variables in every model. Relative risk ratios (RRRs) were calculated for sex-specific OR ratios, and we also computed population-attributable fractions (PAFs) for emerging VAs [16]. Within this research, the PAF represents an estimation of the VAs incidence that might be averted by eradicating the risk determinants. For PAF estimations, continuous data points like LOS-ICU and LODS rating were segmented, using thresholds of 3 and 11, respectively. Purifying the MIMIC-IV database data is pivotal to bolster result accuracy. Illogical or extreme data points were substituted with average figures. Metrics with over 30% omissions were disregarded. For data gaps below 5% of the total count, we applied average value imputation. For data gaps ranging between 5% and 30%, multiple imputations were executed.

Every statistical evaluation was bi-directional, with a *p*-value less than 0.05 deemed to indicate statistical relevance. The statistical computations were carried out using the R software package (version 4.0.4, R Foundation for Statistical Computing, Vienna, Austria) and Stata (version 15.0, StataCorp, College Station, TX, USA).

## 3. Results

### 3.1 Baseline Characteristics

This study recruited 27,139 patients diagnosed with sepsis for analysis (mean [SD] age, 66.6 [16.2] years; 15,626 [57.6%] male). The methodology of the study is detailed in **Supplementary Fig. 1**. We observed that males had a higher prevalence of comorbidities associated with VAs, including CHF, AF, AMI, OMI, NICM, and CKD. Conversely, a larger percentage of females had pneumonia compared to their male counterparts. Furthermore, the use of vasoactive agents and anti-arrhythmic drugs (AAD) was higher in men than in women. Finally, the baseline WBC levels were similar between the two groups (Table 1).

**Table 1.** **Patient characteristics**.

Variables	Total	Women	Men	*p *value
	(n = 27,139)	(n = 11,513)	(n = 15,626)	
Age, years	66.6 ± 16.2	68.3 ± 16.6	65.5 ± 15.8	<0.001
ER admission, %	13,060 (48.1)	5847 (50.8)	5666 (36.3)	<0.001
LOS-ICU, days	4.66 ± 6.07	4.64 ± 5.96	4.68 ± 6.15	0.564
LODS score	5.54 ± 3.43	5.48 ± 3.44	5.58 ± 3.43	0.010
CHF, %	9191 (33.9)	4117 (35.8)	5074 (32.5)	<0.001
VAs, %	1136 (4.2)	348 (3.0)	788 (5.0)	<0.001
AF, %	9760 (36.0)	3974 (34.5)	5786 (37.0)	<0.001
AMI, %	2609 (9.6)	1028 (8.9)	1581 (10.1)	0.001
OMI, %	3769 (13.9)	1323 (11.5)	2446 (15.7)	<0.001
NICM, %	1142 (4.2)	352 (3.1)	790 (5.1)	<0.001
CKD, %	7745 (28.5)	3041 (26.4)	4704 (30.1)	<0.001
ICD, %	259 (1.0)	47 (0.4)	212 (1.3)	<0.001
Pneumonia, %	10,206 (37.6)	4408 (38.3)	5798 (37.1)	0.047
Antibiotics, %	21,878 (80.6)	9301 (80.8)	12,577 (80.5)	0.538
Vasoactive agents, %	12,606 (46.4)	5023 (43.6)	7583 (48.5)	<0.001
AAD, %	5052 (18.6)	1844 (16.0)	3208 (20.5)	<0.001
WBC, × ${\mathrm{10}}^{9}$	11.0 ± 6.1	11.1 ± 6.1	11.1 ± 6.0	0.710

ER, emergency room; LOS-ICU, length of stay in the Intensive Care Units; LODS, logistic organ dysfunction system; CHF, congestive heart failure; VAs, ventricular arrhythmias; AF, atrial fibrillation; AMI, acute myocardial infarction; OMI, old myocardial infarction; NICM, non-ischemic cardiomyopathy; CKD, chronic kidney disease; ICD, implantable cardioverter-defibrillator; AAD, anti-arrhythmia drugs; WBC, white blood cell.

### 3.2 Incidence of VAs in Patients Diagnosed with Sepsis

**Fig. 1.**
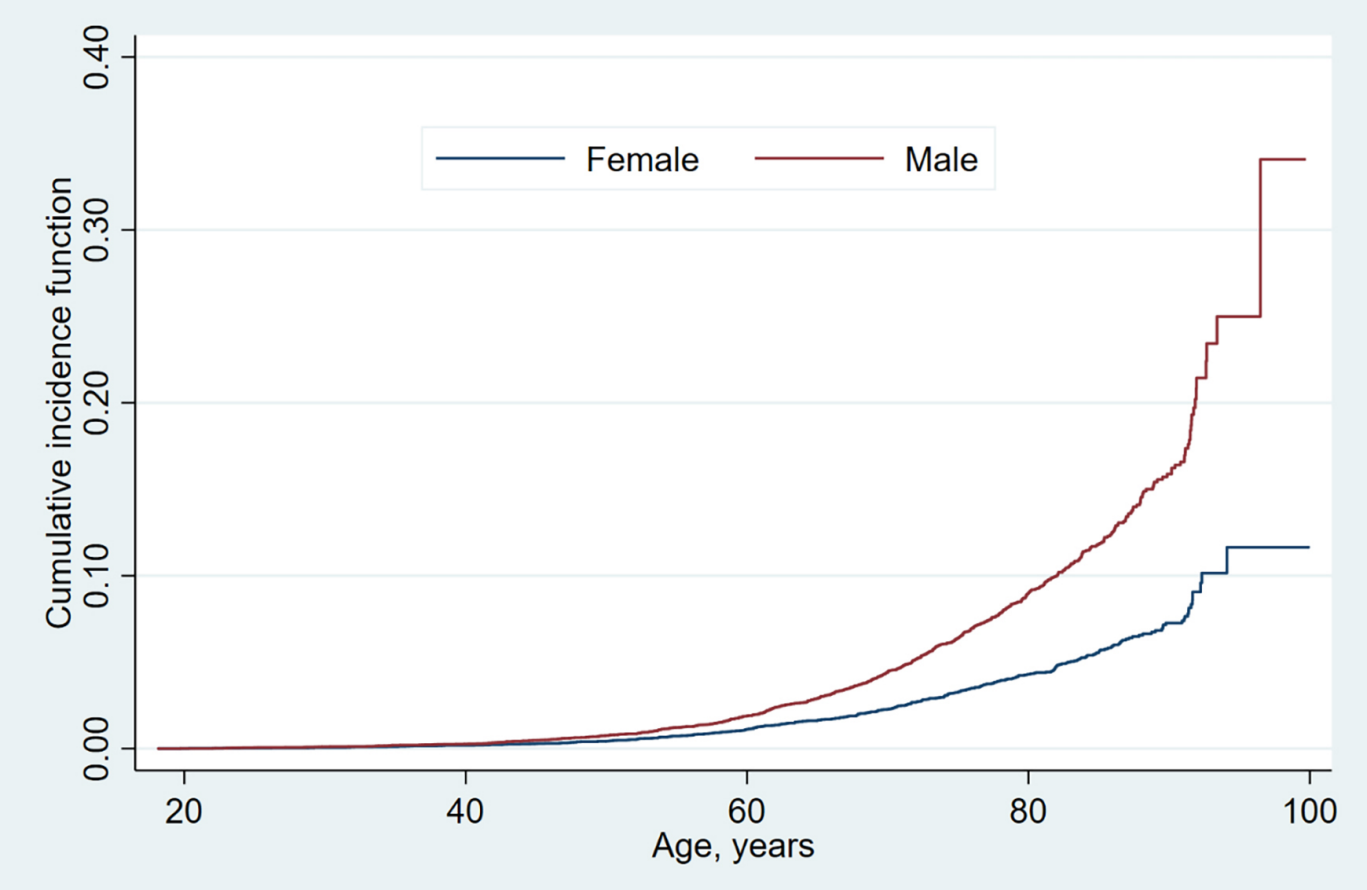
**The cumulative incidence curve for VAs**. Among patients diagnosed with sepsis, VAs were significantly more common in males. Males exhibited an increase in VA incidence after the age of 50, with a similar increase seen in females after the age of 60. VAs, ventricular arrhythmias.

Among patients hospitalized with a simultaneous diagnosis of sepsis, 1136 (4.2%) displayed signs of VAs (VT/VF). A smaller proportion of females exhibited VAs compared to their male counterparts (3.0% vs. 5.0%, *p* < 0.001). The cumulative incidence graph for VAs, considering mortality as a competing factor, can be seen in Fig. 1, demonstrating sex-based differences. For males, a pronounced surge in VAs incidence was evident beyond the age of 50, whereas for females, this uptick was noticeable after 60 years. Prior to reaching 50, both sexes had a relatively low rate of VAs. Beyond 50 years, the rate of VAs escalated with advancing age.

### 3.3 Risk Factors of VAs

In the initial singular logistic regression assessment, all variables, with the exception of age, were correlated with VAs. This finding prompted a more detailed multivariate logistic regression analysis, as detailed in **Supplementary Table 2**. Additionally, **Supplementary Table 3** presents the unadjusted odds ratios (ORs) for VAs based on sex, alongside the interaction *p* values for all the factors under consideration. We found significant sex disparities in the relationship between specific risk factors and VA occurrence. Notably, the interaction *p* values for pneumonia, CHF, and NICM are all less than 0.05, indicating significant interactions with sex. Age demonstrates a differential effect on VAs risk between sexes, with women showing a slightly diminished risk as they age compared to men. Pneumonia significantly elevates the risk of VAs in men but not in women. Additionally, both CHF and NICM exhibit distinct VAs risk associations between men and women. A multivariate model was created utilizing OR as the effect measure. Table 2 presents the ORs adjusted for VAs by sex and the associated interaction *p*-values. We noted a robust link between CHF and an elevated likelihood of VAs across both sexes, with a significant sex-based interaction. Within the multivariate framework, factors like ER admission, LOS-ICU, CKD, and pneumonia did not significantly influence VA occurrence. Additionally, NICM had a marked correlation with VAs, more so in males (OR: 4.072, 95% CI: 3.338–4.967, *p* < 0.001) than in females (OR: 2.501, 95% CI: 1.767–3.541, *p* < 0.001). This resulted in a RRR of 1.628 (95% CI: 1.103–2.403, interaction *p* = 0.014). The relationship between pneumonia and VAs was also noteworthy, with men showing a stronger correlation (OR: 1.132, 95% CI: 0.966–1.326, *p* = 0.124) than women (OR: 0.851, 95% CI: 0.679–1.065, *p* = 0.159), and an interaction *p* value of 0.036. Although in the multivariate analysis, OMI was a risk factor for VAs occurrence in men (OR: 1.365, 95% CI: 1.145–1.627, *p* < 0.001) and not in women (OR: 1.134, 95% CI: 0.859–1.495, *p* = 0.375), the association between OMI and VAs did not show a significant statistical difference between the sexes. An age-corrected Logistic model can be found in **Supplementary Table 4**.

**Table 2.** **Adjusted odds ratios for ventricular arrhythmias risk determinants in the entire cohort, differentiated by sex and accompanied by interaction *p* values**.

Variables	Interaction *p* value	Sex	Odd ratio	*p* value	Relative risk ratio
ER admission	0.945	Men	0.82 (0.71–0.96)	0.014	1.01 (0.77–1.32)
		Women	0.82 (0.65–1.02)	0.073	
LOS-ICU	0.848	Men	1.00 (0.99–1.02)	0.530	1.00 (0.98–1.02)
		Women	1.01 (0.99–1.02)	0.504	
LODS score	0.286	Men	1.06 (1.03–1.08)	<0.001	1.02 (0.98–1.06)
		Women	1.03 (1.00–1.07)	0.032	
CHF	0.031	Men	2.27 (1.90–2.71)	<0.001	1.35 (1.03–1.76)
		Women	1.68 (1.34–2.13)	<0.001	
AF	0.080	Men	1.06 (0.91–1.24)	0.463	0.79 (0.60–1.03)
		Women	1.35 (1.08–1.68)	0.009	
AMI	0.931	Men	2.95 (2.49–3.51)	<0.001	1.01 (0.76–1.36)
		Women	2.92 (2.28–3.74)	<0.001	
OMI	0.249	Men	1.37 (1.15–1.63)	<0.001	1.20 (0.88–1.65)
		Women	1.13 (0.86–1.50)	0.375	
NICM	0.014	Men	4.07 (3.34–4.97)	<0.001	1.63 (1.10–2.40)
		Women	2.50 (1.77–3.54)	<0.001	
CKD	0.506	Men	0.97 (0.83–1.15)	0.757	1.10 (0.83–1.45)
		Women	0.89 (0.70–1.12)	0.315	
Pneumonia	0.036	Men	1.13 (0.97–1.33)	0.124	1.33 (1.02–1.74)
		Women	0.85 (0.68–1.07)	0.159	
Vasoactive agents	0.619	Men	1.27 (1.08–1.50)	0.004	1.07 (0.82–1.40)
		Women	1.19 (0.95–1.49)	0.137	
Antibiotics	0.495	Men	2.15 (1.62–2.87)	<0.001	1.18 (0.73–1.90)
		Women	1.83 (1.24–2.69)	0.002	
WBC	0.874	Men	1.02 (1.00–1.03)	0.013	1.00 (0.98–1.02)
		Women	1.02 (1.00–1.03)	0.049	

ER, emergency room; LOS-ICU, length of stay in the Intensive Care Units; LODS, logistic organ dysfunction system; CHF, congestive heart failure; AF, atrial fibrillation; AMI, acute myocardial infarction; OMI, old myocardial infarction; NICM, non-ischemic cardiomyopathy; CKD, chronic kidney disease; WBC, white blood cell.

### 3.4 PAFs of Risk Factors

Table 3 showcases the PAFs for VAs in inpatient settings, stemming from possible risk determinants. Most risk elements exhibited similar PAFs across both sexes. However, distinctions were observed in a few variables. In particular, the PAF for CHF in males (PAF 16.5%, 95% CI: 12.9–19.7) exceeded that of females (PAF 11.0%, 95% CI: 5.4–15.9). A similar trend was seen in NICM, where men had a PAF of 17.5% (95% CI: 14.4–20.5) compared to women’s 7.4% (95% CI: 3.8–10.8). In contrast, AF showed a higher PAF in women (PAF 15.9%, 95% CI: 6.3–24.5) than in men (PAF 3.0%, 95% CI: –6.7–6.8).

**Table 3.** **Percentage of population-attributable fraction for in-hospital ventricular arrhythmias occurrences, differentiated by sex**.

Variables	PAF (95% CI) Men	PAF (95% CI) Women
ER admission	–7.6 (–13.6–0.1)	–7.4 (–18.1–2.2)
LOS-ICU ≥3	12.2 (5.1–18.9)	7.0 (–3.7–16.6)
LODS ≥11	5.3 (2.5–8.1)	3.6 (–3.7–4.3)
CHF	16.5 (12.9–19.7)	11.0 (5.4–15.9)
AF	3.0 (–6.7–6.8)	15.9 (6.3–24.5)
AMI	9.8 (8.0–11.8)	10.5 (7.8–31.2)
OMI	6.4 (2.0–10.7)	2.9 (–2.8–8.2)
NICM	17.5 (14.4–20.5)	7.4 (3.8–10.8)
CKD	–1.6 (–8.1–4.5)	–0.9 (–9.5–7.0)
Pneumonia	4.6 (–2.3–10.1)	–5.3 (–15.4–4.0)
Antibiotics	5.4 (3.4–6.9)	5.7 (3.0–7.7)
Vasoactive agents	10.4 (2.4–17.7)	10.2 (–9.7–20.2)
WBC ≥10	7.7 (9.7–13.8)	7.1 (–3.7–16.8)

ER, emergency room; LOS-ICU, length of stay in the Intensive Care Units; LODS, logistic organ dysfunction system; CHF, congestive heart failure; AF, atrial fibrillation; AMI, acute myocardial infarction; OMI, old myocardial infarction; NICM, non-ischemic cardiomyopathy; CKD, chronic kidney disease; WBC, white blood cell.

### 3.5 Mortality of VAs

**Fig. 2.**
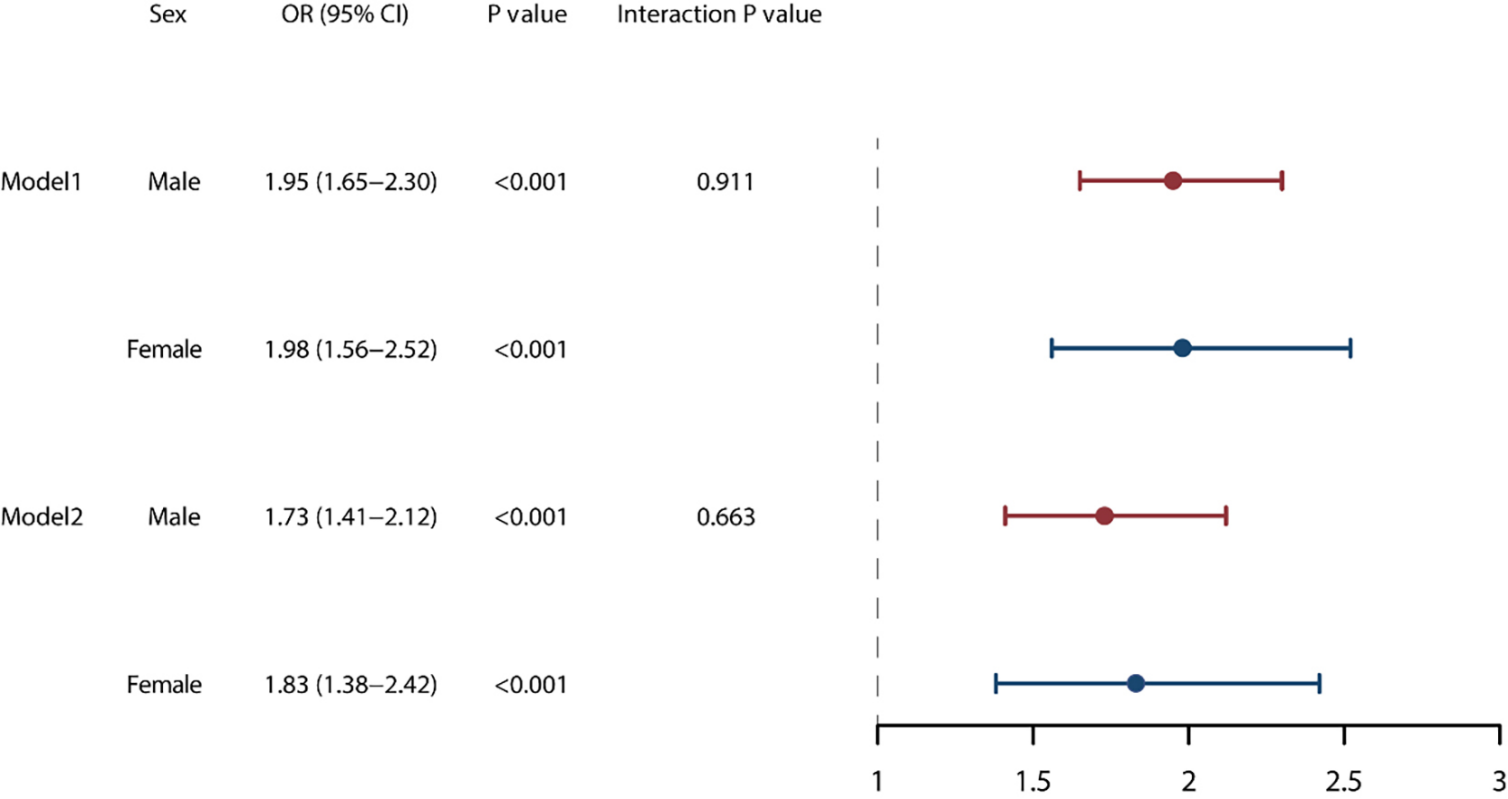
**In-hospital mortality risk models between the sexes**. Model 1 includes adjustments for age-adjusted, while Model 2 incorporates adjustments for various factors including admission pattern, LODS score, LOS-ICU, comorbidities such as CHF, AF, AMI NICM, OMI, and CKD. Additionally, it accounts for pneumonia, the use of vasoactive agents and antibiotics, and WBC count. LOS-ICU, length of stay in the Intensive Care Units; LODS, logistic organ dysfunction system; CHF, congestive heart failure; AF, atrial fibrillation; AMI, acute myocardial infarction; OMI, old myocardial infarction; NICM, non-ischemic cardiomyopathy; CKD, chronic kidney disease; WBC, white blood cell.

**Fig. 3.**
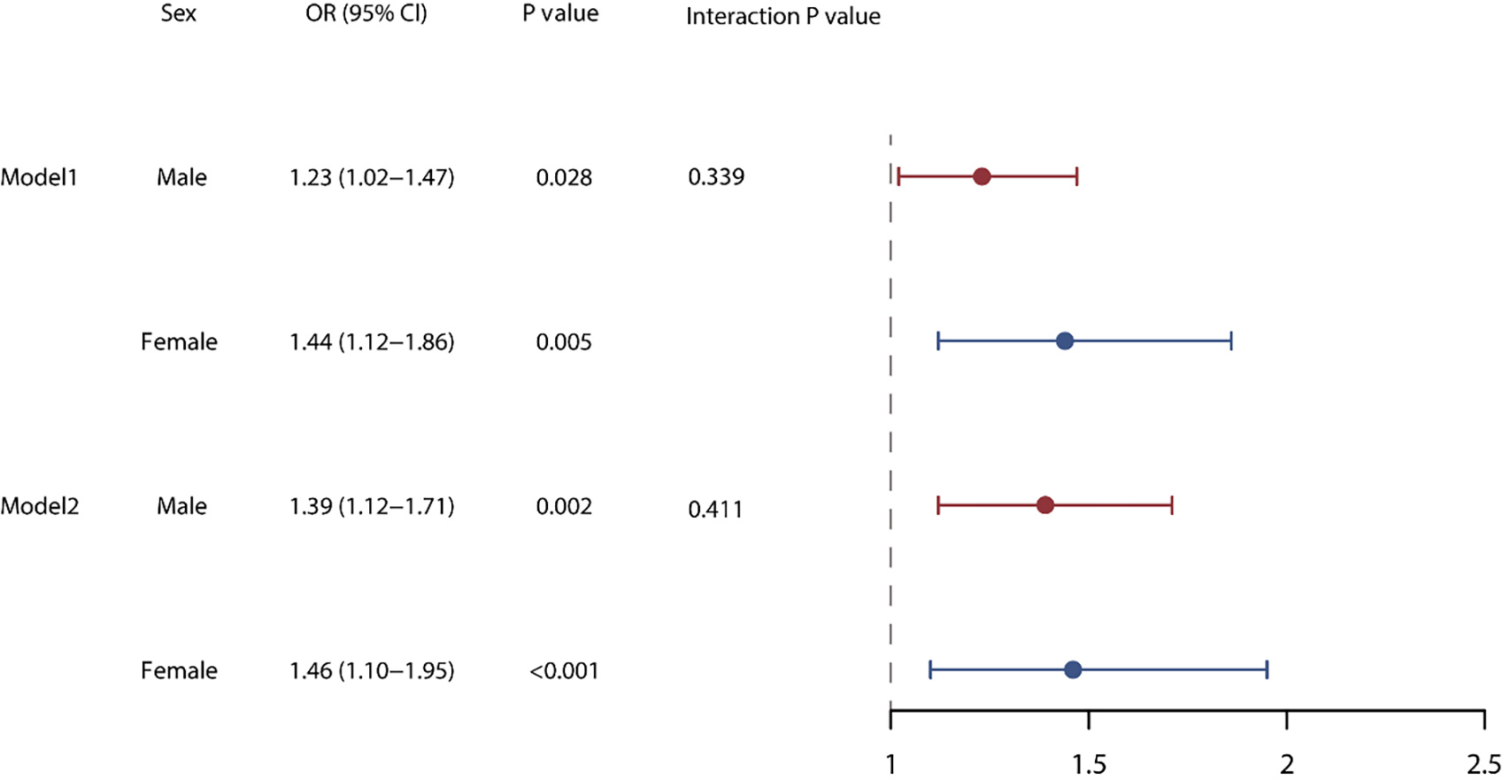
**Mortality risk for VA within one year of treatment, differences between the different sexes**. Model 1 includes adjustments for age-adjusted, while Model 2 incorporates adjustments for various factors including admission pattern, LODS score, LOS-ICU, comorbidities such as CHF, AF, AMI NICM, OMI, and CKD. Additionally, it accounts for pneumonia, the use of vasoactive agents and antibiotics, and WBC count. VA, ventricular arrhythmia; LOS-ICU, length of stay in the Intensive Care Units; LODS, logistic organ dysfunction system; CHF, congestive heart failure; AF, atrial fibrillation; AMI, acute myocardial infarction; OMI, old myocardial infarction; NICM, non-ischemic cardiomyopathy; CKD, chronic kidney disease; WBC, white blood cell.

The age adjusted model (Model 1) identified that VAs almost doubled the mortality risk during hospitalization for both sexes. After adjusting for risk factors (Model 2) VAs emerged as independent risk factors for in-hospital death, regardless of sex. The interplay between in-hospital death and VAs, based on sex, was not significant in either model, with interaction *p* values exceeding 0.05 (Fig. 2). Furthermore, we found that the occurrence of VAs during hospitalization influenced the long-term prognosis of septic patients, leading to an almost 1.5 times heightened risk of mortality within a year. However, there was no significant difference between sexes (interaction *p* values > 0.05) (Fig. 3).

## 4. Discussion

In this large-scale cohort study, we observed sex disparities in VAs incidence and risk factors. With age as the metric, men had a higher VAs risk, particularly in older age groups. The multivariate model showed a stronger link between NICM, CHF, and VAs in men. Furthermore, the occurrence of VAs during hospitalization was associated with a nearly 2-fold increased risk of in-hospital mortality in septic patients, and this risk was consistent across both sexes.

Despite the underrepresentation of female patients in VAs-focused randomized controlled trials, existing research underscores distinct lifetime risks of VAs and SCD between the sexes [7]. This underrepresentation in clinical trials may be linked to the lower incidence of coronary artery disease and the frequency of VAs in women compared to men [7]. In our study, we found that 4.2% of hospitalized sepsis patients experienced VAs. Notably, men had a significantly higher incidence of VAs than women. Furthermore, being male was found to be a strong predictor of increased VAs risk in sepsis. Additionally, Santangeli *et al*. [17] reported that women with heart failure (HF) exhibit a lower rate of receiving appropriate implantable cardioverter-defibrillator (ICD) shocks in comparison to men. The underlying mechanisms that lead to VAs in sepsis remain incompletely understood. Several factors, such as electrolyte disturbance, inflammation, oxidative stress, cardiomyocyte apoptosis, exotoxins, endotoxins and ischemic heart disease are believed to play pivotal roles [18, 19, 20, 21]. The sex disparities observed might be attributed to the effects of sex hormones on ${\mathrm{Ca}}^{2+}$ currents [22, 23].

Our findings reveal that for sepsis patients, the cumulative incidence of VAs remains particularly low up until the age of 50. After this age, there’s a marked increase in incidence for men, whereas for women, this increase is observed after 60 years. Interestingly, we observed a decade-long delay in the onset of VAs for women compared to men, a pattern consistent with prior studies [14]. This decade-long difference in VAs onset between sexes might be attributed to protective hormonal factors in women that diminish post-menopause. As patients approach the age of 95, the incidence rates for both sexes tend to stabilize. Previous studies have established that increasing age is a potent predictor for the onset of VAs, a relationship that was also evident in our current research [5, 24]. Furthermore, the role of CHF as a risk factor for VAs has been previously established, which aligns with our study results [14, 25]. Notably, we observed that CHF has a more pronounced impact on men than on women. Potential mechanisms underlying the occurrence of VAs in CHF patients encompass structural and mechanical alterations in the ventricles, ventricular metabolic abnormalities, electrophysiological changes, neurohormonal imbalances, and the use of vasoactive agents [26, 27]. Moreover, the relationship between CHF and VAs is bidirectional, potentially creating a vicious cycle that exacerbates CHF progression and further heightens susceptibility to VAs [28]. Future prospective studies should further validate these sex differences and elucidate their underlying mechanisms. Risk factors such as NICM, which includes hypertrophic cardiomyopathy (HCM) and NICM, play a crucial role in evaluating patient susceptibility to for VAs and SCD [29, 30]. Our study clearly showcased sex disparities. However, contrasting findings were reported in the Multicenter Automatic Defibrillator Implantation Trial With Cardiac Resynchronization Therapy (MADIT-CRT) [31]. The authors indicated that there was no statistically significant difference in the incidence of VAs and SCD between males and females within NICM (*p* = 0.063) [31]. Interestingly, the cumulative incidence of VAs was lower in females compared to males, a pattern consistent with our study [31]. It’s noteworthy to mention that in this research, female participants constituted only 35% of the total cohort. It’s important to highlight that other investigations into sex differences in VAs incidence are constrained by a limited number of female participants.

Additionally, among sepsis patients who developed VAs, the impact of AF might be more pronounced in females compared to males. There is growing evidence suggesting a mechanistic connection between AF and ventricular tachyarrhythmias [7, 32]. This connection may be attributed to reduced ventricular refractoriness and the occurrence of pro-arrhythmic short-long-short sequences preceding the onset of ventricular tachyarrhythmias in the presence of AF, compared to when the heart is in sinus rhythm [32]. The sex disparity might be related to the fact that female patients often do not receive timely treatment for AF [7]. Furthermore, while our study identified sex differences in the impact of pneumonia on the occurrence of VAs, pneumonia does not appear to be a critical influencing factor for VAs development. A prior study regarding COVID-19 indicated that cardiac arrest and arrhythmias might result from systemic illness rather than being solely a direct consequence of COVID-19 infection [33]. The influence of LOS-ICU and CKD on VAs risk seems limited. For these factors, our study did not observe a significant sex effect on the occurrence of VAs during hospitalization in sepsis patients. However, our study did not incorporate CKD staging, creatinine clearance, or renal replacement therapy in the analysis, so the impact of CKD on VAs should be interpreted with caution.

At present, there remains a debate regarding sex differences in the mortality rate associated with VAs [34]. Our study indicates that the occurrence of VAs in sepsis is correlated with an elevated in-hospital mortality rate and a heightened one-year mortality rate, yet no significant sex disparity was observed. Variations in cardiac regulation, cellular electrophysiology, and the influence of sex hormones might explain the sex-based differences in arrhythmia [7, 34]. Our study unveils the intricate interplay of various risk factors in determining the likelihood of VAs occurrence in sepsis patients. The observed sex disparities in VAs incidence and associated risks underscore the importance of adopting a sex-specific approach in clinical assessments and interventions. Future research should place greater emphasis on potential sex differences within certain cardiovascular diseases.

## 5. Conclusions

Our investigation delineates the nuanced interplay between sepsis and VAs, underscoring salient sex-based disparities in both incidence and predisposing factors. Our data reveals an augmented predisposition to VAs in male patients, with determinants such as CHF and NICM exerting a more pronounced influence in this demographic. The etiological underpinnings of these observations are intricate, spanning cellular electrophysiology, cardiac autoregulation, and the modulatory effects of sex hormones. These insights advocate for a sex-centric paradigm in clinical evaluations and therapeutic interventions. Emphasizing this, it becomes crucial to advocate for sophisticated, sex-tailored therapeutic strategies in addressing these challenges.

## 6. Limitations

While this cohort study benefits from a substantial population size, several limitations warrant mention. First, the study’s retrospective design necessitates the need for future prospective investigations. Second, the absence of baseline electrocardiograms in this cohort may have resulted in an underestimation of VA incidence. Third, the data in this article originates from a substantial intensive care medicine cohort, which may limit the generalizability of our findings to other patient populations. Additionally, we did not account for iatrogenic VAs in our analysis despite their relatively frequent occurrence in ICU settings. Given the limitations of our data source, we are unable to account for iatrogenic VAs in our analysis. Studying iatrogenic VAs and their relationship with sex among ICU patients is indeed a valuable topic that merits further research. Lastly, the method used for imputing missing may have influenced our results. In light of these limitations, the conclusions drawn from this study should be approached with prudence.

## Data Availability

The datasets analyzed during the current study are available from the corresponding author on reasonable request.

## Supplementary Material

Supplementary material associated with this article can be found, in the online version, at https://doi.org/10.31083/j.rcm2504132.
