# Supplementary material for: Sex Differences in the Epidemiology, Risk Factors, and Prognosis of Malignant Ventricular Arrhythmias in Sepsis Patients
Source: Rev Cardiovasc Med. 2024 Apr 3;25(4):132. doi: 10.31083/j.rcm2504132 (PMC11263981; doi:10.31083/j.rcm2504132)
Supplement: Supplementary file 1 [file 2153-8174-25-4-132-s1.docx]

**Supplementary Table 1.** Definitions of the included variables.

| **Variables** | **Definitions** |
| --- | --- |
| Sepsis | Sepsis was defined as infection-related organ dysfunction which was represented by an increase in the Sequential Organ Failure Assessment (SOFA) score of 2 points or more. |
| VA | Including non-sustained and sustained (> 30s) ventricular tachycardia, and ventricular fibrillation. All diagnoses of VA are based on ECGs. |
| LODS score | The first evaluation of LODS score after admission is selected. |
| CHF | Heart failure with symptoms of congestion. |
| AF | An ECG strip with ≥ 30s of AF, regardless of types of AF. |
| AMI | Including ST elevation and non-ST elevation of myocardial infarction. |
| OMI | A history of myocardial infarction > 3 months. |
| NICM | Including non-ischemic dilated cardiomyopathy, alcoholic cardiomyopathy, hypertrophic cardiomyopathy, and diabetic cardiomyopathy. |
| CKD | Including CKD stage 1 to 5. |
| Pneumonia | Including hospital-acquired pneumonia and community-acquired pneumonia |
| Antibiotics | Including macrolide and quinolone antibiotics. Regardless of dosage. |
| Vasoactive agents | Including epinephrine, norepinephrine, phenylephrine, dopamine, and dobutamine. Regardless of dosage. |
| AAD | Including propafenone, amiodarone, dronedarone, and sotalol. Regardless of dosage. |

VA: ventricular arrhythmia; LODS: Logistic organ dysfunction system; SHF: systolic heart failure; SBP: systolic blood pressure; AF: atrial fibrillation; AMI: acute myocardial infarction; OMI: old myocardial infarction; NICM: non-ischemic cardiomyopathy; CKD: chronic kidney injury; AAD: anti-arrhythmia drugs.

| Supplementary Table 2. Non–adjusted odd ratios for ventricular arrhythmia risk factors in the overall sample | | |
| --- | --- | --- |
| Variables | Odd ratio (95% CI) | *P* value |
| Age | 0.99 (0.99–1.00) | 0.294 |
| Male | 1.70 (1.50–1.94) | < 0.001 |
| ER admission | 0.76 (0.67–0.85) | < 0.001 |
| LOS_ICU | 1.02 (1.01–1.03) | < 0.001 |
| LODS score | 1.09 (1.07–1.12) | < 0.001 |
| CHF | 3.64 (3.22–4.12) | < 0.001 |
| AF | 1.91 (1.69–2.15) | < 0.001 |
| AMI | 5.60 (4.92–6.38) | < 0.001 |
| OMI | 2.79 (2.44–3.18) | < 0.001 |
| NICM | 8.35 (7.16–9.74) | < 0.001 |
| CKD | 1.91 (1.69–2.15) | < 0.001 |
| Pneumonia | 1.49 (1.33–1.68) | < 0.001 |
| Antibiotics | 3.03 (2.43–3.79) | < 0.001 |
| Vasoactive agents | 1.71 (1.51–1.93) | < 0.001 |
| WBC | 1.02 (1.01–1.02) | 0.002 |

| Supplementary Table 3. Non–adjusted ventricular arrhythmia odd ratios by sex and interaction P values for ventricular arrhythmia risk factors in the overall sample | | | | | |
| --- | --- | --- | --- | --- | --- |
| Variables | Interaction *P* value | Sex | Odd ratio | *P* value | Relative risk ratio |
| Age | **0.020** | Men | 1.00 (0.99–1.01) | 0.298 | 1.01 (1.00–1.02) |
|  |  | Women | 0.99 (0.99–0.99) | 0.035 |  |
| ER admission | 0.781 | Men | 0.76 (0.66–0.88) | < 0.001 | 0.96 (0.74–1.25) |
|  |  | Women | 0.79 (0.64–0.98) | 0.032 |  |
| LOS_ICU | 0.907 | Men | 1.02 (1.01–1.03) | < 0.001 | 1.00 (0.98–1.02) |
|  |  | Women | 1.02 (1.01–1.03) | 0.007 |  |
| LODS score | 0.253 | Men | 1.09 (1.07–1.12) | < 0.001 | 1.02 (0.99–1.06) |
|  |  | Women | 1.07 (1.04–1.10) | < 0.001 |  |
| CHF | **< 0.001** | Men | 4.33 (3.72–5.04) | < 0.001 | 1.61 (1.24–2.10) |
|  |  | Women | 2.69 (2.17–3.34) | < 0.001 |  |
| AF | 0.329 | Men | 1.80 (1.57–2.09) | < 0.001 | 0.88 (0.68–1.14) |
|  |  | Women | 2.06 (1.66–2.55) | < 0.001 |  |
| AMI | 0.464 | Men | 5.74 (4.90–6.71) | < 0.001 | 1.11 (0.84–1.48) |
|  |  | Women | 5.16 (4.08–6.53) | < 0.001 |  |
| OMI | 0.124 | Men | 2.87 (2.46–3.35) | < 0.001 | 1.27 (0.94–1.72) |
|  |  | Women | 2.26 (1.74–2.93) | < 0.001 |  |
| NICM | **0.007** | Men | 9.08 (7.60–10.21) | < 0.001 | 1.68 (1.15–2.45) |
|  |  | Women | 5.41 (3.89–7.52) | < 0.001 |  |
| CKD | 0.106 | Men | 2.00 (1.73–2.31) | < 0.001 | 1.24 (0.95–1.62) |
|  |  | Women | 1.61 (1.29–2.01) | < 0.001 |  |
| Pneumonia | **0.027** | Men | 1.65 (1.43–1.90) | < 0.001 | 1.34 (1.03–1.74) |
|  |  | Women | 1.23 (0.99–1.53) | 0.061 |  |
| Vasoactive agents | 0.436 | Men | 1.73 (1.49–2.00) | < 0.001 | 1.11 (0.86–1.44) |
|  |  | Women | 1.56 (1.26–1.93) | < 0.001 |  |
| Antibiotics | 0.295 | Men | 3.30 (2.51–4.35) | < 0.001 | 1.28 (0.81–2.05) |
|  |  | Women | 2.58 (1.77–3.75) | < 0.001 |  |
| WBC | 0.691 | Men | 1.01 (1.00–1.03) | 0.019 | 1.00 (0.98–1.02) |
|  |  | Women | 1.02 (1.00–1.03) | 0.034 |  |

| Supplementary Table 4. Age–adjusted ventricular arrhythmia odd ratios by sex and interaction P values for ventricular arrhythmia risk factors in the overall sample | | | | | |
| --- | --- | --- | --- | --- | --- |
| Variables | Interaction *P* value | Sex | Odd ratio | *P* value | Relative risk ratio |
| ER admission | 0.760 | Men | 0.76 (0.66–0.88) | < 0.001 | 0.96 (0.74–1.25) |
|  |  | Women | 0.79 (0.64–0.98) | 0.033 |  |
| LOS_ICU | 0.907 | Men | 1.02 (1.01–1.03) | < 0.001 | 1.00 (0.98–1.02) |
|  |  | Women | 1.02 (1.01–1.03) | 0.008 |  |
| LODS score | 0.257 | Men | 1.10 (1.07–1.12) | < 0.001 | 1.02 (0.99–1.06) |
|  |  | Women | 1.07 (1.04–1.10) | < 0.001 |  |
| CHF | **< 0.001** | Men | 5.01 (4.28–5.86) | < 0.001 | 1.59 (1.22–2.07) |
|  |  | Women | 3.16 (2.53–3.95) | < 0.001 |  |
| AF | 0.302 | Men | 2.05 (1.76–2.39) | < 0.001 | 0.87 (0.68–1.13) |
|  |  | Women | 2.35 (1.88–2.93) | < 0.001 |  |
| AMI | 0.462 | Men | 5.91 (5.05–6.92) | < 0.001 | 1.11 (0.84–1.48) |
|  |  | Women | 5.32 (4.20–6.74) | < 0.001 |  |
| OMI | 0.121 | Men | 2.97 (2.54–3.48) | < 0.001 | 1.27 (0.94–1.72) |
|  |  | Women | 2.34 (1.80–3.03) | < 0.001 |  |
| NICM | **0.006** | Men | 9.13 (7.64–10.91) | < 0.001 | 1.69 (1.16–2.46) |
|  |  | Women | 5.41 (3.89–7.53) | < 0.001 |  |
| CKD | 0.091 | Men | 2.11 (1.82–2.45) | < 0.001 | 1.26 (0.96–1.64) |
|  |  | Women | 1.68 (1.34–2.10) | < 0.001 |  |
| Pneumonia | **0.027** | Men | 1.65 (1.43–1.90) | < 0.001 | 1.34 (1.03–1.74) |
|  |  | Women | 1.23 (0.99–1.53) | 0.060 |  |
| Vasoactive agents | 0.427 | Men | 1.73 (1.49–2.01) | < 0.001 | 1.11 (0.86–1.44) |
|  |  | Women | 1.56 (1.26–1.93) | < 0.001 |  |
| Antibiotics | 0.292 | Men | 3.31 (2.51–4.35) | < 0.001 | 1.29 (0.81–2.05) |
|  |  | Women | 2.57 (1.76–3.75) | < 0.001 |  |
| WBC | 0.688 | Men | 1.01 (1.00–1.03) | 0.020 | 1.00 (0.98–1.02) |
|  |  | Women | 1.02 (1.00–1.03) | 0.034 |  |


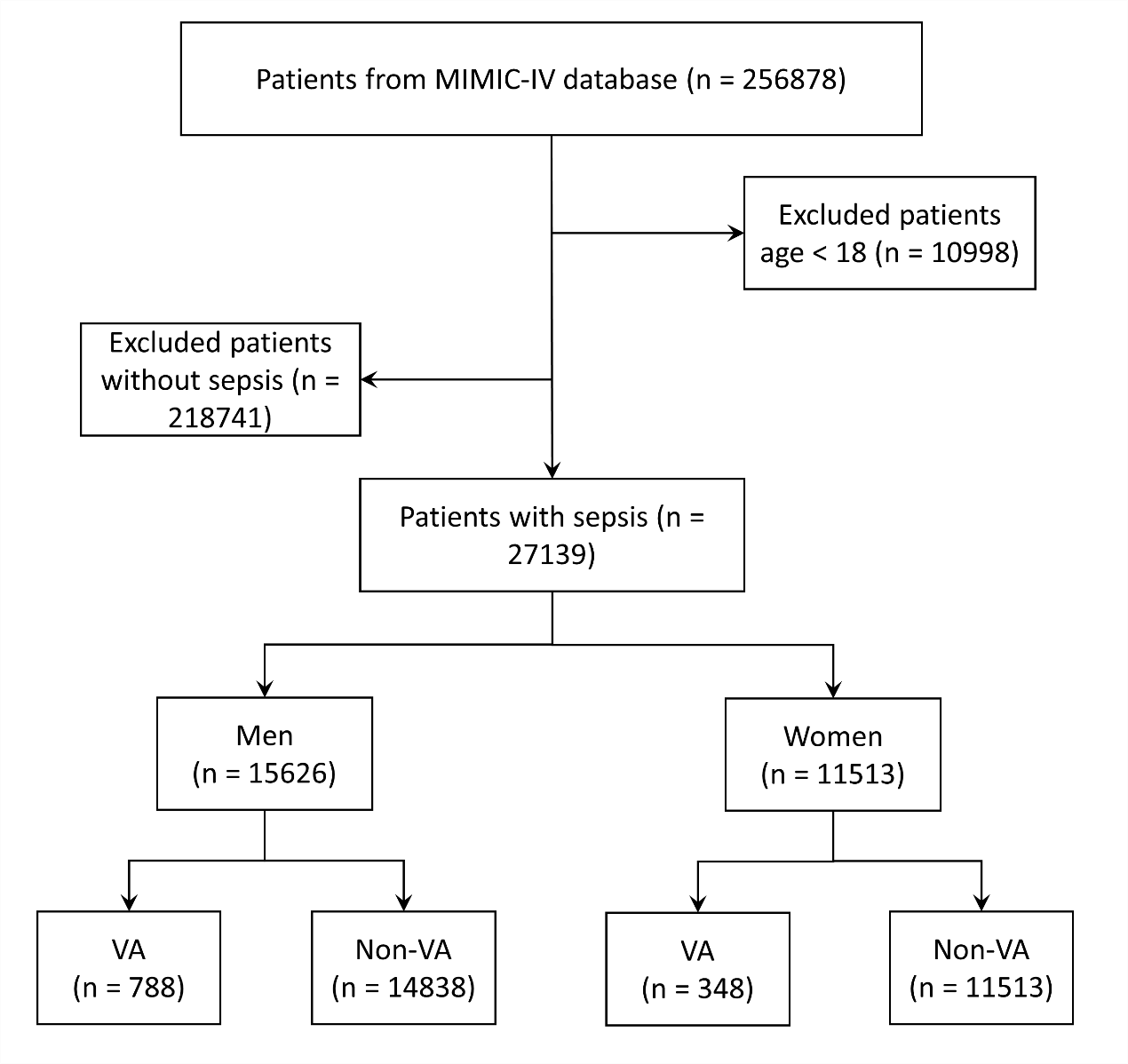


**Supplementary Fig. 1**. Flow chart.
